# Molecular characterization and virulence gene profiling of methicillin-resistant *Staphylococcus aureus* associated with bloodstream infections in southern China

**DOI:** 10.3389/fmicb.2022.1008052

**Published:** 2022-10-17

**Authors:** Rui Zhao, Xing Wang, Xinhui Wang, Bingyu Du, Kexin Xu, Faming Zhang, Changhong Jiang, Yanfeng Zhao, Yefei Zhu

**Affiliations:** ^1^Laboratory Medicine Center, The Second Affiliated Hospital, Nanjing Medical University, Nanjing, China; ^2^Department of Laboratory Medicine, Shanghai Children’s Medical Center, Shanghai Jiao Tong University School of Medicine, Shanghai, China; ^3^Medical Center for Digestive Diseases, The Second Affiliated Hospital, Nanjing Medical University, Nanjing, China; ^4^Department of Laboratory Medicine, Zhujiang Hospital, Southern Medical University, Guangzhou, China

**Keywords:** bloodstream infection, methicillin-resistant *Staphylococcus aureus*, sequence typing, virulence genes, antibiotic resistance

## Abstract

Methicillin-resistant *Staphylococcus aureus* (MRSA) causes an enormous illness burden, including skin and soft tissue infections (SSTIs), pneumonia, bloodstream infections (BSI), and sepsis. BSI are associated with significant patient morbidity and mortality worldwide. However, limited information is available on MRSA-related BSI in China. This study aimed to investigate the molecular characterization of 77 MRSA isolates recovered from hospitalized patients with BSI between 2012 and 2020 at three first-class tertiary hospitals in southern China based on multilocus sequence typing (MLST), *spa* typing, and staphylococcal cassette chromosome mec (SCC*mec*) typing. Overall, 13 clonal complexes (CCs) were identified, with CC59 and CC5 being the largest clusters, indicating high genetic diversity among BSI-causing MRSA isolates. ST59 was the most prevalent MLST type (22.1%). ST5/ST764-MRSA SCC*mec* II was the predominant adult MRSA clone, whereas ST59-MRSA SCC*mec* IV was the most common pediatric MRSA clone. ST5-t2460, ST764-t1084, and ST59-t437 were the most common types of adult MRSA isolates, whereas ST59-t437 and ST59-t172 were the predominant types of children’s MRSA isolates. ST59-SCC*mec* IV/V represented the most common clone among community acquired-MRSA isolates. ST5/ST764-SCC*mec* II was the most common type of hospital-associated MRSA isolate. The most prevalent toxin-encoding genes detected were *hla*, *hld*, *icaA*, and *clfA* (96.1–100%). Forty-three (100%, 43/43) isolates harbored more than 18 of the tested virulence genes in adults and eight virulence genes (23.5%, 8/34) in children. Virulence gene analysis revealed diversity among different clones: the positivity rates for the Panton-Valentine leukocidin (PVL) gene were 55.8 and 35.3% in adult and pediatric MRSA isolates, respectively; the genes *seb–sei* were present in all adult strains; *seb–seg–sei–seo* were present in all ST5, ST59, ST15, ST45, and ST22 adult strains; and *seg–sei–sem–sen–seo* were present in different clones, including ST15, ST45, and ST22 adult MRSA isolates and ST25, ST30, ST546, and ST72 children’s MRSA isolates. Adult MRSA isolates had significantly higher antibiotic resistance rates and virulence gene prevalence than pediatric MRSA isolates. For 8 years, this study provided epidemiological data on the molecular characteristics and virulence genes in different groups of MRSA BSI in China. Our findings may provide critical information for a better understanding of MRSA BSI.

## Introduction

The spread of *Staphylococcus aureus*, an important opportunistic pathogen both in healthcare and community settings, is a universal challenge. Since the 1960s, methicillin-resistant *Staphylococcus aureus* (MRSA) has emerged, disseminated globally, and become a leading cause of bacterial infections in communities and hospitals (Lee et al., 2018). *S. aureus* causes a large burden of diseases, such as skin and soft tissue infections (SSTIs), pneumonia, postoperative infections, bloodstream infections (BSI), sepsis, biofilm-associated infections, and bacterial endocarditis (Lowy, 1998), which places a tremendous burden on health care services and carries enormous consequences for societies and economies. Furthermore, BSI have been associated with significant patient morbidity and mortality worldwide (Mcnamara et al., 2018). In a BSI organism study, among the 264,901 BSI isolates collected, the most common pathogens were *S. aureus* and *Escherichia coli* (Diekema et al., 2019). In a recent study, MRSA BSI, particularly those with delayed culture clearance, was associated with high mortality (Johnson et al., 2021). These findings are significant because MRSA exhibits resistance to multiple antibiotics (except β-lactams) and possesses different exotoxin gene profiles. Additionally, the primary cause of *S. aureus* infections being persistent and challenging to eradicate is biofilm formation (Lister and Horswill, 2014).

The genotype of *S. aureus* has been reported to influence the severity, complications, and mortality of infection. Currently, various molecular subtyping approaches have been developed for the characterization of *S. aureus*, such as the staphylococcal chromosomal cassette *mec* (SCC*mec*) typing, multilocus sequence typing (MLST), and staphylococcal protein A (*spa*) typing. In China, ST59-MRSA-t437-IV is the most common type among children and adolescents (Li et al., 2014; Yang et al., 2017). In a study of MRSA isolated from children, t437 (65%) was the most prevalent, followed by t441 (6.7%). Approximately 85% of isolates harbored SCC*mec* type IV, followed by SCC*mec* V (10%), whereas no isolates harbored SCC*mec* I, II, and III (Yang et al., 2017). The predominant clonal complex (CC) and *spa* type were CC59 (32.7%) and t172 (16.8%), respectively, and ST59-SCC*mec* IV-t172/t437 was the most prevalent epidemic clone among children in Shanghai (Song et al., 2017). Furthermore, 34 (42.5%) isolates harbored more than 10 tested virulence genes (Wang et al., 2016). It has been reported that bacteria adhesion is a critical first step in biofilm formation (Foster et al., 2014; Lister and Horswill, 2014). Adhesion genes were found in most of the MRSA isolates obtained from children, which included *icaA* (100%), *clfA* (100%), *sdrC* (95%), and *sdrE* (63.8%) (Wang et al., 2016).

Over the past few decades, new MRSA clones have been discovered. In contrast to earlier studies where MRSA was found to almost exclusively occur in hospitalized patients, the new clones can invade community settings and ultimately infect people with no predisposing risk factors (Lakhundi and Zhang, 2018). As reported in previous studies, various community-acquired MRSA (CA-MRSA) strains have been discovered circulating in different countries or regions. ST80-IV clones were mainly found in Europe, ST1-IV and ST8-IV clones were more prevalent in the United States and Canada, and ST59-IV/V were the most common CA-MRSA clones in China and several other Asian countries (Wang et al., 2016). Another study reported that the ST59 CA-MRSA clone was circulating in Taiwan and other areas worldwide (Huang and Chen, 2011). In Europe, all isolates related to the *spa* type t032 were MRSA (Ludden et al., 2015). Additionally, the *spa* type t037 in Africa and t037 and t437 in Australia consisted exclusively of MRSA isolates (Asadollahi et al., 2018). CA-MRSA isolates harbored different types of SCC*mec* elements when compared with conventional hospital-associated MRSA (HA-MRSA) strains. To date, 13 SCC*mec* types (indicated by Roman numerals I to XIII) and three mec (*mecA/B/C*) genes have been identified worldwide among MRSA strains (Hiramatsu et al., 2013; Kaya et al., 2018). HA-MRSA infections are typically associated with SCC*mec* types I, II, and III, whereas CA-MRSA clones are associated with SCC*mec* types IV and V, which are smaller cassettes lacking resistance genes to non-beta-lactam antibiotics (Lakhundi and Zhang, 2018).

It has been reported that MRSA developed resistance to most antibiotics used to effectively treat and control its infections, where CA-MRSA was resistant to fewer antibiotics than HA-MRSA (Lakhundi and Zhang, 2018). Generally, CA-MRSA is more virulent than HA-MRSA because of the presence of extremely varying virulence factors (Deurenberg and Stobberingh, 2008). The increased expression of various genetic components has also been associated with increased virulence in CA-MRSA (Otto, 2013). For instance, an important cytotoxin secreted by *S. aureus* is Panton-Valentine leukocidin (PVL), which is encoded by *lukS-PV* and *lukF-PVG* (Genestier et al., 2005). Moreover, PVL is harbored by most CA-MRSA strains and is rarely found in HA-MRSA strains in Europe and the United States (Deleo et al., 2010); however, situations differed in some regions of China (Bai et al., 2021). The positivity rates for the staphylococcal enterotoxin (SE) genes, arginine catabolic mobile gene (*arcA*), leukocidin gene (*lukE*), hemolysin genes (*hla*, *hlb*, *hld*, *hlg*, and *hlg2*), and adhesion genes (*clfA*, *icaA*, *sdrC*, *sdrD*, and *sdrE*) were found to be closely related to different types of MRSA isolates. Further, the distribution of some virulence genes, particularly enterotoxin genes, was found to be related to different MRSA lineages. CC1 and CC5 isolates were found to harbor more enterotoxin genes than other CC isolates, indicating that different CCs had different virulence profiles (Diep et al., 2006; Kim et al., 2011). However, the significance of these virulence factors in MRSA bacteremia is not well elucidated.

To understand the toxicity, molecular characteristics, and antimicrobial resistance of these strains, it is critical to analyze a few significant genetic elements and perform sequence typing. This study, therefore, aimed to investigate the molecular profiles, associated virulence genes, and drug resistance of 77 MRSA isolates recovered from BSI inpatients between 2012 and 2020 in three first-class tertiary hospitals in southern China.

## Materials and methods

### Collection and detection of microbial specimens

Convenience samples of MRSA strains were collected from three first-class tertiary hospitals. Twenty-six nonrepeating CA-MRSA isolates were obtained from pediatric patients with BSI (<18 years old) in Shanghai Children’s Medical Center from July 2012 to February 2017. Additionally, 25 nonduplicate MRSA strains were collected at the Second Affiliated Hospital of Nanjing Medical University, and 26 MRSA isolates were collected at Zhujiang Hospital of Southern Medical University (from 2016 to 2020). All *S. aureus* strains were isolated from the culture of blood samples of inpatients with BSI. MRSA isolates were confirmed through antibiotic susceptibility testing, and the presence of *mecA* was confirmed using polymerase chain reaction (PCR) as previously described. CA-MRSA was defined using the Centers for Disease Control and Prevention (CDC) criteria, whereas HA-MRSA was defined using the Ministry of Health’s Diagnostic Criteria for Nosocomial Infections.

### Antibiotic susceptibility testing

There were 16 drugs tested: penicillin (P), oxacillin (OXA), erythromycin (E), clindamycin (DA), ciprofloxacin (CIP), levofloxacin (LVX), moxifloxacin (MOF), tetracycline (TET), gentamicin (GM), rifampicin (RF), trimethoprim–sulfamethoxazole (STX), quinupristin/dalfopristin (Q/D), linezolid (LZD), vancomycin (V), tigecycline (TGC), and cefoxitin (FOX). *S. aureus* ATCC 29213 was used for quality control in drug resistance research. Antibiotic resistance spectra of all MRSA isolates were generated using the bioMérieux VITEK2 system according to the manufacturer’s instructions. The recommendations and definitions provided in the current Clinical and Laboratory Standards Institute guidelines were used to interpret drug sensitivities.

### *Spa* typing

Staphylococcus protein A (*spa*) is a 40–60 kDa surface protein with three components: Fc-partial-region, X-region, and C-terminal. *Spa* typing is a genotyping method in which the polymorphic X-region based on a variable number of 24-bp repeat sequences is amplified and sequenced. DNA amplification products were analyzed for Sanger sequencing using primers 1113f (5′-TAAAGACGATCCTTCGGTGAGC-3′) and 1514r (5′-CAGCAGTAGTGCCGTTTGCTT-3′) according to the protocol described in the official Ridom *Spa* Server website.^1^ After the sequences were evaluated, *spa* types were assigned using the Ridom StaphType™ software (Ridom GmbH, Würzburg, Germany).^3^

### MLST analysis

Multilocus sequence typing, a sequence-based genotyping method, was performed as previously reported by [Enright et al., 2000)]. The sequences of the seven (approximately 450-bp long) internal fragments of housekeeping genes—*arcC*, *aroE*, *glpF*, *gmk*, *pta*, *tpi*, and *yqi*—were amplified using seven respective PCR assays and sequenced using Sanger dideoxy DNA sequencing. The assignment of analyzed sequences and the determination of sequence types (STs) and CCs were performed by comparing the sequences of the PCR products to those of the existing alleles available from the MLST database.^2^ Clustering of related STs that were defined as cloned CCs was performed using the eBURST (Based Upon Related Sequence types) algorithm.

### SCC*mec* typing

Boye et al. (2007) described SCC*mec* typing of MRSA isolates, which was based on a series of multiplex PCR reactions using eight specific primers. In our study, eight primers were designed manually and purchased commercially. All oligonucleotide primers used in this study were synthesized by Sangon Biotech (Shanghai, China). SCC*mec* types I–V were identified based on the combination of the cassette chromosome recombinase (*ccr*) type and *mec* class. Amplified PCR products were visualized on a 1.2% agarose gel stained with ethidium bromide under UV transillumination. All primers are listed in Supplementary Table S1.

### Detection of virulence genes

The following 35 MRSA virulence genes were identified using PCR: SE genes (*sea*, *seb*, *sec*, *sed*, *see*, *seg*, *seh*, *sei*, *sej*, *sel*, *sem*, *sen*, *seo*, *sep*, *seq*, and *sek*) (Zhang et al., 2018), arginine catabolic mobile gene (*arcA*), toxic shock syndrome toxin-1 gene (*tst*), exfoliative toxin genes (*eta* and *etb*), leukocidin genes (*lukF/S-PV*, *lukE*, and *lukM*) (Lina et al., 1999; Yamada et al., 2005), bacteriocin gene (*bsaA*), hemolysin genes (*hla*, *hlb*, *hld*, *hlg*, and *hlg2*), adhesion genes (*clfA*, *icaA*, *sdrC*, *sdrD*, and *sdrE*), and *EDIN*, as previously described (Arvidson and Tegmark, 2001; Jarraud et al., 2002; Peacock et al., 2002; Bubeck Wardenburg et al., 2007). The virulence genes were detected and amplified using an S1000 thermal cycler (Bio-Rad, Hercules, California, United States) under the following conditions: an initial denaturation for 5 min at 94°C, followed by 32 cycles of 45 s at 94°C, 30 s at 60°C, and 1 min at 72°C. The final elongation was 7 min at 72°C. Amplified PCR products were separated *via* 1.2% agarose gel electrophoresis in 1× TAE buffer at 110 V for 42 min. The gel was stained with ethidium bromide and exposed to UV light for visualizing the amplified products. When the bands were unclear, the experiment was repeated several times to confirm reproducibility.

### Statistical analysis

GraphPad Prism 9.0 (GraphPad Software Inc., San Diego, CA, United States) and IBM SPSS Statistics (SPSS Inc., Chicago, IL, United States) were used to statistically analyze the data and create graphs. The Chi-square test (χ^2^) was used to analyze categorical data. A *p* value of <0.05 was considered statistically significant.

## Results

### MLST, *spa*, and SCC*mec* typing

Multilocus sequence typing was used to examine the evolutionary and genetic diversity of 77 MRSA isolates obtained from patients with BSI (Table 1). Overall, 26 STs were identified among the MRSA strains, which were clustered using eBURST into 13 CCs. The largest clusters were CC59 and CC5, each with 22 and 21 isolates, respectively. These were followed by CC398 with 10 isolates, CC8 with 6 isolates, CC1 with 5 isolates, CC45 and CC88 with 3 isolates each, CC15 with 2 isolates, and CC22, CC25, CC30, CC72, and CC188 with 1 isolate each. The most prevalent ST was ST59 (22.1%, 17/77), followed by ST5 (11.7%, 9/77), ST764 (10.4%, 8/77), and ST398 (9.1%, 7/77). The genetic diversity of the isolates was confirmed using *spa* typing. A total of 32 *spa* types were observed, and three isolates could not be assigned to any known type. The most predominant type was *spa* t437 (14.3%, 11/77).

**Table 1:** Molecular characteristics and antibiotic resistance profiles of 77 bloodstream infection methicillin-resistant *S. aureus* (MRSA) obtained from inpatients.

		CCs MLST (n, %)	*spa* type (n, %)	SCC*mec* type (n, %)	Antimicrobial resistance (R %)
Adults	CC5	ST5 (9, 11.7%)	t2460 (4, 5.2%), t002 (2, 2.6%),	II (18, 23.4%), IV (2, 2.6%)	p (100), OXA (100), E (100), DA (100)
			t1084 (2, 2.6%), t311 (1, 1.3%)		CIP (100), LVX (100), MOF (100)
		ST764 (7, 9.1%)	t1084 (4, 5.2%), t002 (2, 2.6%),		TET (90), GM (75), FOX (100)
			t111 (1, 1.3%)		
		ST6290 (2, 2.6%)	t1084 (2, 2.6%)		
		ST6 (1, 1.3%)	t4298 (1, 1.3%)		
		ST5985 (1, 1.3%)	t311 (1, 1.3%)		
	CC59	ST59 (4, 5.2%)	t437 (4, 5.2%)	IV (4, 5.2%), V (4, 5.2%)	P (100), OXA (100), E (100)
		ST338 (2, 2.6%)	t3590 (1, 1.3%), t3595 (1, 1.3%)		DA (100), TET (12.5), FOX (100)
		ST951 (2, 2.6%)	t437 (2, 2.6%)		
	CC8	ST239 (3, 3.9%)	t037 (3, 3.9%)	III (4, 5.2%), V (1, 1.3%)	P (100), OXA (100), E (80), DA (80)
					CIP (100), LVX (100), MOF (100)
		ST6570 (1, 1.3%)	t2196 (1, 1.3%)		TET (80), GM (80), RF (20), STX (60)
		ST7212 (1, 1.3%)	t030 (1, 1.3%)		FOX (100)
	CC398	ST398 (2, 2.6%)	t034 (2, 2.6%)	V (4, 5.2%), III (1, 1.3%)	P (100), OXA (100), E (40), DA (40)
					CIP (20), LVX (20), MOF (20), TET (20)
		ST6697 (2, 2.6%)	t034 (1, 1.3%), t8616 (1, 1.3%)		GM (20), FOX (100)
		ST6285 (1, 1.3%)	t034 (1, 1.3%)		
	CC15	ST15 (2, 2.6%)	t084 (1,1.3%), NT^a^	IV (2, 2.6%)	P (100), OXA (100), E(50), DA (50), FOX (100)
	CC45	ST45 (2, 2.6%)	t116 (1, 1.3%), t1768 (1, 1.3%)	IV (2, 2.6%)	P (100), OXA (100), E (50), DA (50)
					CIP(50), LVX(50), RF(50), FOX(100)
	CC22	ST22 (1, 1.3%)	t309 (1, 1.3%)	I (1, 1.3%)	P(100), OXA(100), E(100), DA(100)
					FOX(100)
Children	CC59	ST59 (13, 16.9%)	t437 (4, 5.2%), t172 (4, 5.2%),	IV (9, 11.7%), V (4, 5.2%), III (1, 1.3%)	P(100), OXA(100), E(78.6), DA(14.3)
			t441 (3, 3.9%)		TET(7.1), FOX(100)
			t163 (1, 1.3%), t3485 (1, 1.3%)		
		ST951 (1, 1.3%)	t437 (1, 1.3%)		
	CC1	ST1 (4, 5.2%)	t114 (2, 2.6%), t177 (2, 2.6%)	IV (5, 6.5%)	P(100), OXA(100), E(80), DA(60)
		ST5904 (1, 1.3%)	t177 (1, 1.3%)		FOX(100)
	CC398	ST398 (5, 6.5%)	t034 (3, 3.9%), t1446 (1, 1.3%)	V (4, 5.2%), IV (1, 1.3%)	P(100), OXA(100), E(40), DA(20), FOX(100)
			NT^a^		
	CC88	ST88 (3, 3.9%)	t2310 (1, 1.3%), t12147 (1, 1.3%) NT^a^	V (2, 2.6%), IV (1, 1.3%)	*p*(100), OXA(100), E(66.7), DA(66.7), LVX (33.3), STX(33.3), FOX(100)
	CC5	ST764 (1, 1.3%)	t1084 (1, 1.3%)	II (1, 1.3%)	P(100), OXA(100), E(100), DA(100)
					CIP(100), LVX(100), MOF(100), TET(100), FOX(100)
	CC8	ST630 (1, 1.3%)	t4549 (1, 1.3%)	V (1, 1.3%)	P(100), OXA(100), E(100), CIp(100)
					LVX(100), MOF(100), TET(100), GM(100), RF(100), FOX(100)
	CC25	ST25 (1, 1.3%)	t349 (1, 1.3%)	?	P(100), OXA(100), E(100), DA(100), FOX(100)
	CC30	ST30 (1, 1.3%)	t019 (1, 1.3%)	IV (1, 1.3%)	P(100), OXA(100), FOX(100)
	CC45	ST546 (1, 1.3%)	t116 (1, 1.3%)	IV (1, 1.3%)	P(100), OXA(100), FOX(100)
	CC72	ST72 (1, 1.3%)	t148 (1, 1.3%)	?	P(100), OXA(100), FOX(100)
	CC188	ST188 (1, 1.3%)	t2769 (1, 1.3%)	IV (1, 1.3%)	P(100), OXA(100), FOX(100)

a Nt, MRSA isolates that could not be assigned to any expected type were defined as non-typable (NT).

Using SCC*mec* typing, five types (I, II, III, IV, and V) were identified among the 77 MRSA isolates. The most predominant type of *S. aureus* was type IV (37.7%, 29/77), accounting for approximately one-third of all MRSA isolates, followed by V (26%, 20/77), II (24.7%, 19/77), III (7.8%, 6/77), and I (1.3%, 1/77). MRSA isolates that could not be assigned to any expected type were defined as nontypable (NT), and two isolates were classified as NT for SCC*mec* typing.

The combination of CCs and *spa* types yielded the following predominant combinations: CC59-t437 (14.3%, 11/77), CC5-t1084 (11.7%, 9/77), and CC398-t034 (9.1%, 7/77). A strong association was observed between certain STs and *spa* types. ST59 was primarily associated with t437 (47.1%, 8/17), ST764 was associated mainly with t1084 (62.5%, 5/8), and ST398 was associated mainly with t034 (71.4%, 5/7). However, when the CCs and SCC*mec* types were combined, the predominant combinations were CC5-SCC*mec* II (24.7%, 19/77) and CC59-SCC*mec* IV (16.9%, 13/77). CC5 was primarily associated with SCC*mec* II (90.5%, 19/21), CC398 was associated mainly with V (80%, 8/10), and CC1 was associated mainly with IV (100%, 5/5). A strong association was also observed between certain STs and SCC*mec* types. Among all isolates of ST5/ST764-MRSA, the predominant SCC*mec* type was type II (Table 1).

### Virulence gene profiles and *mecA* gene

A total of 35 putative virulence genes were detected. Table 2 lists the frequencies of the virulence genes identified in the 77 MRSA isolates. All MRSA isolates harbored seven or more virulence genes. The most prevalent toxin-encoding genes detected were *hla* (100%), *hld* (100%), *icaA* (100%), and *clfA* (96.1%). Among all MRSA isolates, the positivity rates for *lukM* (0%), *EDIN* (0%), *eta* (0%), *etb* (0%), *arcA* (1.3%), and *see* (5.2%) were low. Additionally, *hlb*, *hlg*, *hlg2*, *sdrC*, *sdrD*, *sdrE*, *lukE*, *seb*, *sei*, *seg*, *sek*, *sel*, *sem*, *sen*, *seo*, and *seq* were detected in 58.4–92.2% of the isolates, whereas the remaining ones were found in <50% of the isolates.

**Table 2:** Frequencies of virulence and enterotoxin genes among the molecular types of 77 bloodstream infection methicillin-resistant *S. aureus* (MRSA) obtained from inpatients.

	CCs	Virulence genes detected (n, %)	Enterotoxin genes detected (n, %)
Adults	CC5	*sdrC(18,90), sdrD(18,90), sdrE(19,95), arcA(1,5)*	*sea(13,65),seb(20,100),sec(8,40),seg(20,100),seh(11,55)*
		*icaA(20,100)clfA(18,90), TssT-1 (9,45)*	*sei(20,100),sej(13,65),sek(14,70),sel(18,90),sem(17,85)*
		*lukE(20,100), pvl(9,45), bsaA(4,20), hla(20,100), hlb(13,65)*	*sen(17,85),seo(20,100),sep(2,10),seq(11,55)*
		*hld(20,100), hlg(18,90)hlg2 (20,100)*	
	CC59	*sdrC(8,100),sdrD(7,87.5),sdrE(6,75),icaA(8,100)clfA(8,100)*	*sea(2,25),seb(8,100),sec(1,12.5),see(1,12.5)seg(8,100)*
		*lukE(8,100), pvl(6,75), bsaA(2,25), hla(8,100), hlb(8,100)*	*seh(5,62.5),sei(8,100),Sej(6,75),sek(8,100),sel(8,100),*
		*hld(8,100), hlg(6,75)hlg2 (8,100)*	*sem(7,87.5),sen(8,100),seo(8,100),sep(2,25),seq(7,87.5)*
	CC8	*sdrC(3,60), sdrD(4,80), sdrE(5,100), icaA(5,100), clfA(4,80)*	*sea(5,100),seb(5,100),sec(1,20),seg(5,100),seh(4,80)*
		*TssT-1 (1,20), lukE(5,100), pvl(1,20), bsaA(5,100)*	*sei(5,100),sej(4,80),sek(5,100),sel(5,100),sem(4,80)*
		*hla(5,100), hlb(4,80)hld(5,100), hlg(5,100)hlg2 (5,100)*	*sen(4,80),seo(4,80),sep(1,20),seq(5,100)*
	CC398	*sdrC(5,100), sdrD(5,100), sdrE(5,100), icaA(5,100)clfA(5,100)*	*sea(3,60),seb(5,100),sec(1,20),seg(4,80),seh(1,20)*
		*TssT-1 (1,20), lukE(5,100), pvl(5,100), bsaA(1,20), hla(5,100)*	*sei(5,100),sej(4,80),sek(5,100),sel(5,100),sem(5,100)*
		*hld(5,100), hlg(5,100)hlg2 (4,80)*	*sen(4,80),seo(4,80),seq(3,60)*
	CC15	*sdrC(2,100),sdrD(2,100),sdrE(2,100),icaA(2,100)clfA(2,100)*	*seb(2,100),seg(2,100),seh(2,100),sei(2,100)*
		*lukE(2,100),pvl(2,100),hla(2,100),hld(2,100)*	*sej(2,100),sek(2,100),sel(2,100),sem(2,100)*
		*hlg(2,100),hlg2 (2,100)*	*sen(2,100),seo(2,100)sep(1,50),seq(1,50)*
	CC45	*sdrC(1,50),sdrD(1,50),sdrE(1,50),icaA(2,100)clfA(2,100),*	*seb(2,100),sec(1,50),seg(2,100),seh(2,100),sei(2,100)*
		*lukE(2,100),hla(2,100),hld(2,100),hlg(2,100)*	*sej(2,100),sek(1,50),sel(2,100),sem(2,100),sen(2,100)*
			*seo(2,100),seq(1,50)*
	CC22	*sdrC(1,100),sdrD(1,100),sdrE(1,100),icaA(1,100)clfA(1,100)*	*sea(1,100),seb(1,100),seg(1,100),sei(1,100)*
		*lukE(1,100),pvl(1,100),bsaA(1,100),hla(1,100),hld(1,100)*	*sek(1,100),sel(1,100),sem(1,100),sen(1,100),seo(1,100)*
		*hlg(1,100),hlg2 (1,100)*	*sep(1,100),seq(1,100)*
Children	CC59	*sdrC(13,92.9),sdrD(3,21.4),sdrE(14,100),icaA(14,100)*	*sea(8,57.1),seb(14,100),sec(2,14.3),sed(4,28.6),seg(3,21.4)*
		*clfA(14,100),lukE(10,71.4),pvl(6,42.9),bsaA(1,7.1),hla(14,100)*	*she(2,14.3),sei(2,14.3),sej(1,7.1),sek(14,100),sel(2,14.3)*
		*hlb(14,100),hld(14,100),hlg(3,21.4)hlg2 (14,100)*	*sem(2,14.3),sen(2,14.3),seo(2,14.3),seq(13,92.9)*
	CC1	*sdrC(5,100),sdrD(5,100),sdrE(5,100),icaA(5,100)clfA(5,100)*	*seb(3,60),sec(4,80),sed(1,20),seg(3,60),seh(4,80),sei(3,60)*
		*TssT-1 (4,80),lukE(5,100),bsaA(4,80),hla(5,100),hlb(3,60)*	*sej(3,60),sek(5,100),sel(5,100),sem(1,20),sen(2,40)*
		*hld(5,100),hlg(3,60),hlg2 (4,80)*	*seo(2,40),seq(5,100)*
	CC398	*sdrC(5,100),icaA(5,100),clfA(5,100),TssT-1 (1,20),lukE(3,60)*	*sed(2,40)*
		*pvl (1,20),bsaA(3,60),hla(5,100)*	
		*hld(5,100),hlg(5,100),hlg2 (2,40)*	
	CC88	*sdrC(3, 100),sdrD(2,66.7),sdrE(2,66.7),icaA(3, 100)clfA(3,100)*	*sea(2,66.7),seb(1, 33.3),sec(2,66.7),sed(2,66.7),see(3, 100)*
		*lukE(3, 100),pvl(2,66.7)*	*sep(3, 100)*
		*hla(3, 100),hlb(2,)hld(3, 100),hlg2 (3, 100)*	
	CC5	*sdrE(1,100), icaA(1,100),clfA(1,100)*	*sea(1,100),seb(1,100),seg(1,100),seh(1, 100),sei(1, 100)*
		*TssT-1 (1,100),lukE(1,100),pvl(1,100),bsaA(1,100),*	*sej(1, 100),sek(1, 100),sel(1, 100),sen(1, 100),seo(1, 100)*
		*hla(1,100),hld(1,100),hlg(1,100),hlg2 (1,100)*	*seq(1, 100)*
	CC8	*sdrC(1,100),icaA(1, 100)clfA(1,100)*	*sed(1, 100)*
		*lukE(1,100)hla(1,100),hlb(1,100)*	
		*hld(1,100),hlg2 (1,100)*	
	CC25	*sdrC(1,100),sdrD(1, 100),sdrE(1, 100)*	*seb(1, 100),sed(1, 100),seg(1, 100),sei(1, 100),*
		*icaA(1, 100),clfA(1, 100),lukE(1, 100),pvl(1, 100)*	*sem(1, 100),sen(1, 100),seo(1, 100)*
		*bsaA(1, 100),hla(1, 100),hlb(1, 100),hld(1, 100),hlg2 (1, 100)*	
	CC30	*sdrC(1,100), icaA(1, 100)clfA(1, 100)*	*seg(1, 100),sei(1, 100),sem(1, 100),sen(1, 100),seo(1, 100)*
		*lukE(1, 100),pvl(1, 100) hla(1, 100),hld(1,100)*	
		*hlg(1, 100),hlg2 (1, 100)*	
	CC45	*sdrC(1, 100),sdrE(1, 100),icaA(1, 100)clfA(1, 100),*	*sea(1, 100),seb(1, 100),sec(1, 100), seg(1, 100),seh(1, 100)*
		*lukE(1, 100),bsaA(1, 100)*	*sei(1, 100),sej(1, 100),sek(1, 100),sel(1, 100),*
		*hla(1, 100),hld(1, 100),hlg(1, 100)*	*sem(1, 100),sen(1, 100),seo(1, 100),seq(1, 100)*
	
	CC72	*sdrC(1, 100),sdrD(1, 100), icaA(1, 100)clfA(1, 100)*	*sea(1, 100),sed(1, 100),seg(1, 100),sei(1, 100),sem(1, 100)*
		*lukE(1, 100)hla(1, 100),hld(1, 100),hlg2 (1, 100)*	*sen(1, 100), seo(1, 100)*
	CC188	*sdrC(1, 100),sdrE(1, 100), icaA(1, 100)*	*sea(1, 100), seb(1, 100), sec(1, 100), sed(1, 100)*
		*clfA(1, 100),lukE(1, 100),hla(1, 100),hlb(1, 100)*	*sek(1, 100), seq(1, 100)*
		*hld(1, 100),hlg2 (1, 100)*	

Sixteen classical enterotoxin genes (*sea*, *seb*, *sec*, *sed*, *see*, *seg*, *seh*, *sei*, *sej*, *sel*, *sem*, *sen*, *seo*, *sep*, *seq*, and *sek*) were detected among the 77 MRSA strains (Table 2). Each enterotoxin gene was found in multiple *S. aureus* isolates, accounting for 5.2–84.4% of the isolates. All 77 strains were used to determine the presence of *mecA*. The results revealed that all of the isolates were *mecA*-positive, and they were therefore classified as MRSA isolates.

### Molecular characteristics of adult and Children’s MRSA isolates

Genetic diversity was observed in MRSA isolates obtained from adults and children. The most common cluster among the adult MRSA isolates was CC5 (26%, 20/77), whereas that among the pediatric isolates was CC59 (18.2%, 14/77). Furthermore, ST5 (11.7%, 9/77) and ST764 (9.1%, 7/77) isolates were mainly found among adult MRSA specimens, whereas ST59 (16.9%, 13/77) and ST398 (6.5%, 5/77) isolates were the most prevalent among pediatric MRSA specimens (Table 1). Additionally, t1084 (10.4%, 8/77) was the predominant *spa* type among the adult MRSA isolates, followed by t437 (7.8%, 6/77) and t2460, t002, and t034 (each with four isolates). Among the pediatric isolates, the most predominant *spa* type was t437 (6.5%, 5/77; Table 1). The most common SCC*mec* type among the adult MRSA isolates was type II, accounting for approximately one-half of all adult MRSA isolates (41.9%, 18/43), whereas types IV, V, III, and I were found in 10, 9, 5 and 1 isolates, respectively. The most predominant SCC*mec* type among pediatric MRSA isolates was type IV (55.9%, 19/34; Table 1), whereas types V, II, and III were found in 11, 1, and 1 isolates, respectively. All five SCC*mec* types were detected in adult MRSA isolates, but type I was not found in children’s MRSA isolates. ST5-t2460 (44.4%, 4/9), ST764-t1084 (57.1%, 4/7), and ST59-t437 (100%, 4/4) were the most common adult MRSA strains, whereas ST59-t437 (30.8%, 4/13) and ST59-t172 (30.8%, 4/13) were the most common pediatric MRSA strains. ST5-SCC*mec* II (100%, 9/9) and ST764-SCC*mec* II (100%, 7/7) were the predominant adult MRSA clones, whereas ST59-SCC*mec* IV (61.5%, 8/13) was the predominant pediatric MRSA clone.

The frequencies of *hlg* (90.7% vs. 41.2%), *sdrD* (88.4% vs. 35.3%), *sdrE* (90.7% vs. 73.5%), *lukE* (100% vs. 82.4%), *seb* (100% vs. 64.7%), *sed* (0% vs. 38.2%), *seg* (97.7% vs. 32.4%), *she* (58.1% vs. 23.5%), *sei* (100% vs. 29.4%), *sej* (72.1% vs. 17.6%), *sel* (95.3% vs. 26.5), *sem* (88.4% vs. 20.6%), *sen* (88.4% vs. 26.5%), and *seo* (95.3% vs. 26.5%) were significantly higher among the adult MRSA isolates than among the pediatric isolates (*p* < 0.05; Table 3). However, no significant difference was observed in the likelihood of other virulence genes between the adult and children’s MRSA isolates (*p* > 0.05). *sed* was only found in pediatric MRSA isolates, whereas *arcA* was only detected in adult MRSA isolates. Table 2 shows that 43 (100%, 43/43) isolates harbored more than 18 tested virulence genes in adults and 8 (23.5%, 8/34) in children. The presence of SE genes was strongly associated with the MLST profile and age. Pediatric MRSA isolates CC398 and CC8 contained only *sed*, but *sed* was not detected in adult MRSA isolates. Virulence gene analysis revealed diversity among different clones: the genes *see–sep* were present only in CC88 pediatric MRSA strains; *seb–sei* were present in all adult strains; *seb–seg–sei–seo* genes were present in all ST5, ST59, ST15, ST45, and ST22 adult strains, whereas *sek* was present in ST59 and ST1 pediatric isolates; and *seg–sei–sem–sen–seo* were present in different clones, including ST15, ST45, and ST22 adult MRSA isolates and ST25, ST30, ST546, and ST72 children’s MRSA strains. The positivity rates for PVL among adult and children’s MRSA isolates were 55.8% (24/43) and 35.3% (12/34), respectively. The PVL gene was detected in 36 strains, which represented 9 different STs, with CC59 being the most common, whereas it was not found in CC45, CC1, or CC398 pediatric isolates. The adhesion gene *icaA*, leukocidin gene *lukE*, and hemolysin genes (*hla*, *hld*, and *hlg2*) were present in all CC5 strains. In addition, all CC59 adult isolates harbored adhesion genes (*clfA*, *icaA*, and *sdrC*), the leukocidin gene *lukE*, and hemolysin genes (*hla*, *hlb*, *hld*, and *hlg2*), whereas all CC59 pediatric isolates harbored *seb–sek–seq*, adhesion genes (*clfA*, *icaA*, and *sdrE*), and hemolysin genes (*hla*, *hlb*, *hld*, and *hlg2*) (Table 2).

**Table 3:** Frequencies of virulence genes among methicillin-resistant *S. aureus* (MRSA) isolates obtained from adults and children.

Virulence genes	*S. aureus (*n, %)	Adults (n, %)	Children (n, %)	*p*-Value^a^
	(*n* = 77)	(*n* = 43)	(*n* = 34)	
*pvl*	36,46.8	24,55.8	12,35.3	0.073
*hla*	77,100	43,100	34,100	
*hlb*	47,61	25,58.1	22,64.7	0.557
*hld*	77,100	43,100	34,100	
*hlg*	53,68.8	39,90.7	14,41.2	*p* < 0.001
*hlg2*	69,89.6	40,93	29,85.3	0.467
*bsaA*	24,31.2	13,30.2	11,32.4	0.842
*icaA*	77,100	43,100	34,100	
*clfA*	74,96.1	40,93	34,100	0.328
*sdrC*	70,90.9	38,88.4	32,94.1	0.637
*sdrD*	50,64.9	38,88.4	12,35.3	*p* < 0.001
*sdrE*	64,83.1	39,90.7	25,73.5	0.046
*lukE*	71,92.2	43,100	28,82.4	0.015
*lukM*	0	0	0	
*arcA*	1,1.3	1,2.3	0	1
*TssT-1*	17,22.1	11,25.6	6,17.6	0.405
*EDIN*	0	0	0	
*eta*	0	0	0	
*etb*	0	0	0	
*sea*	38,49.4	24,55.8	14,41.2	0.202
*seb*	65,84.4	43,100	22,64.7	*p* < 0.001
*sec*	22,28.6	12,27.9	10,29.4	0.885
*sed*	13,16.9	0	13,38.2	*p* < 0.001
*see*	4,5.2	1,2.3	3,8.8	0.448
*seg*	53,68.8	42,97.7	11,32.4	*p* < 0.001
*seh*	33,42.9	25,58.1	8,23.5	0.002
*sei*	53,68.8	43,100	10,29.4	*p* < 0.001
*sej*	37,48.1	31,72.1	6,17.6	*p* < 0.001
*sek*	58,75.3	36,83.7	22,64.7	0.055
*sel*	50,64.9	41,95.3	9,26.5	*p* < 0.001
*sem*	45,58.4	38,88.4	7,20.6	*p* < 0.001
*sen*	47,61	38,88.4	9,26.5	*p* < 0.001
*seo*	50,64.9	41,95.3	9,26.5	*p* < 0.001
*sep*	10,13	7,16.3	3,8.8	0.532
*seq*	50,64.9	29,67.4	21,61.8	0.604

a The positive rates of virulence genes among adult strains were compared to those among children isolates.

### Molecular characteristics of CA-MRSA and HA-MRSA

CA-MRSA and HA-MRSA exhibited considerable genetic diversity. In this study, 28 (36.4%) HA-MRSA and 49 (63.6%) CA-MRSA isolates were analyzed. CC59 and CC5 were the largest clusters with 15 and 10 isolates, respectively, followed by CC398 with 9 isolates, CC8 with 3 isolates, CC88 with 3 isolates, CC45 with 2 isolates, CC1 with 2 isolates, and CC15, CC25, CC30, CC72, and CC188 each with 1 isolate among the CA-MRSA strains. Furthermore, for HA-MRSA, CC5 and CC59 (with 11 and 7 isolates, respectively) were the two dominant types, followed by CC1 with 3 isolates, CC8 with 2 isolates, and CC15, CC22, CC45, and CC398 (each with only one isolate). Further, 12 distinct CCs (20 STs) and 25 *spa* types were identified among CA-MRSA isolates, whereas 8 CCs (15 STs) and 13 *spa* types were found among HA-MRSA strains. The most predominant clone among CA-MRSA isolates was ST59-MRSA (16.9%, 13/77), followed by ST398-MRSA (9.1%, 7/77), ST764-MRSA (6.5%, 5/77), and ST5-MRSA (5.2%, 4/77), whereas, ST5 and ST59 were the two most prevalent MLST types among HA-MRSA isolates. The most common *spa* types among CA-MRSA isolates were t034 (7.8%, 6/77) and t437 (6.5%, 5/77), whereas t1084 (9.1%, 7/77) and t437 (7.8%, 6/77) were the top two *spa* types among HA-MRSA strains. The predominant SCC*mec* types of HA-MRSA were IV and II, whereas IV and V were the most common SCC*mec* types in CA-MRSA (Table 4).

**Table 4:** Molecular characteristics of 77 community-acquired MRSA (CA-MRSA) and hospital-associated MRSA (HA-MRSA) isolates.

	CCs	MLST (n, %)	*spa* type (n,%)	SCC*mec* type (n, %)
CA-MRSA	CC59	ST59 (13, 16.9%)	t437 (4, 5.2%), t172 (4, 5.2%), t441 (3, 3.9%)	IV (10, 13%), V (5, 6.5%)
		ST338 (1, 1.3%)	t163 (1, 1.3%), t3485 (1, 1.3%)	
		ST951 (1, 1.3%)	t3590 (1, 1.3%)	
			t437 (1, 1.3%)	
	CC5	ST764 (5, 6.5%)	t1084 (2, 2.6%), t002 (2, 2.6%), t111 (1, 1.3%)	II (10, 13%)
		ST5 (4, 5.2%)	t2460 (2, 2.6%), t311 (1, 1.3%), t002 (1, 1.3%)	
		ST5985 (1, 1.3%)	t311 (1, 1.3%)	
	CC398	ST398 (7, 9.1%)	t034 (5,6.5%),t1446 (1,1.3%), NT	V (7, 9.1%), III (1, 1.3%), IV (1, 1.3%),
		ST6697 (2, 2.6%)	t034 (1, 1.3%), t8616 (1, 1.3%)	
	CC8	ST239 (1, 1.3%)	t037 (1, 1.3%)	V (2, 2.6%), III (1, 1.3%)
		ST6570 (1, 1.3%)	t2196 (1, 1.3%)	
		ST630 (1, 1.3%)	t4549 (1, 1.3%)	
	CC88	ST88 (3, 3.9%)	t2310 (1,1.3%),t12147 (1,1.3%),NT	V (2, 2.6%), IV (1, 1.3%)
	CC45	ST45 (1, 1.3%)	t116 (1, 1.3%)	IV (2, 2.6%)
		ST546 (1, 1.3%)	t116 (1, 1.3%)	
	CC1	ST1 (2, 2.6%)	t114 (2, 2.6%)	IV (2, 2.6%)
	CC15	ST15 (1, 1.3%)	NT	IV (1, 1.3%)
	CC25	ST25 (1, 1.3%)	t349 (1, 1.3%)	?
	CC30	ST30 (1, 1.3%)	t019 (1, 1.3%)	IV (1, 1.3%)
	CC72	ST72 (1, 1.3%)	t148 (1, 1.3%)	?
	CC188	ST188 (1, 1.3%)	t2769 (1, 1.3%)	IV (1, 1.3%)
HA-MRSA	CC5	ST5 (5, 6.5%)	t1084 (2, 2.6%), t2460 (2, 2.6%), t002 (1, 1.3%)	II (9, 11.7%), IV (2, 2.6%)
		ST764 (3, 3.9%)	t1084 (3, 3.9%)	
		ST6290 (2, 2.6%)	t1084 (2, 2.6%)	
		ST6 (1, 1.3%)	t4298 (1, 1.3%)	
	CC59	ST59 (4, 5.2%)	t437 (4, 5.2%)	IV (3, 3.9%), V (3, 3.9%), III (1,1.3%)
		ST951 (2, 2.6%)	t437 (2, 2.6%)	
		ST338 (1, 1.3%)	t3595 (1, 1.3%)	
	CC1	ST1 (2, 2.6%)	t117 (2, 2.6%)	IV (3, 3.9%)
		ST5940 (1, 1.3%)	t117 (1, 1.3%)	
	CC8	ST239 (2, 2.6%)	t037 (2, 2.6%)	III (3, 3.9%)
		ST7212 (1, 1.3%)	t030 (1, 1.3%)	
	CC15	ST15 (1, 1.3%)	t084 (1, 1.3%)	IV (1, 1.3%)
	CC22	ST22 (1, 1.3%)	t309 (1, 1.3%)	I (1, 1.3%),
	CC45	ST45 (1, 1.3%)	t1768 (1, 1.3%)	IV (1, 1.3%)
	CC398	ST6285 (1, 1.3%)	t034 (1, 1.3%)	V (1, 1.3%)

In this study, the most prevalent clone found among CA-MRSA isolates was ST59-SCC*mec* IV/V. Other common clones were ST764/ST5-SCC*mec* II and ST398-SCC*mec*V. The most common type of HA-MRSA isolate was ST5/ST764-SCC*mec* II. Furthermore, both CA-MRSA and HA-MRSA isolates comprised ST59, ST5, ST764, ST1, ST951, ST15, ST45, ST239, and ST338. *lukE–seb–seg–sei* were present in all HA-MRSA strains. The frequencies of *sdrC*, *sdrD*, *lukE*, *seb*, *sed*, *seg*, *seh*, *sei*, *sej*, *sek*, *sel*, *sem*, *sen*, and *seo* were significantly lower among the CA-MRSA isolates than among the HA-MRSA isolates (*p* < 0.05). However, no significant difference was found in the likelihood of other virulence genes between the CA- and HA-MRSA isolates (*p* > 0.05). The positivity rates for the gene encoding PVL among the CA- and HA-MRSA isolates were 53.1% (26/49) and 35.7% (10/28), respectively (data not shown; Tables 4, 5).

**Table 5:** Prevalence of virulence genes among community-acquired MRSA (CA-MRSA) and hospital-associated MRSA (HA-MRSA) isolates.

	*S*. *aureus*	CA-MRSA	HA-MRSA	*p*-Value^a^
	*n* = 77, n (%)	*n* = 49, n (%)	*n* = 28, n (%)	
*pvl*	36 (46.8)	26 (53.1)	10 (35.7)	0.142
*hla*	77 (100)	49 (100)	28 (100)	
*hlb*	47 (61)	30 (61.2)	17 (60.7)	0.965
*hld*	77 (100)	49 (100)	28 (100)	
*hlg*	53 (68.8)	29 (59.2)	24 (85.7)	0.016
*hlg2*	69 (89.6)	42 (85.7)	27 (96.4)	0.274
*bsaA*	24 (31.2)	13 (26.5)	11 (39.3)	0.245
*icaA*	77 (100)	49 (100)	28 (100)	
*clfA*	74 (96.1)	47 (95.9)	27 (96.4)	1
*sdrC*	70 (90.9)	48 (98)	22 (78.6)	0.015
*sdrD*	50 (64.9)	27 (55.1)	23 (82.1)	0.017
*sdrE*	64 (83.1)	39 (79.6)	25 (89.3)	0.438
*lukE*	71 (92.2)	43 (87.8)	28 (100)	
*lukM*	0	0	0	
*arcA*	1 (1.3)	0	1 (3.6)	0.364
*TssT-1*	17 (22.1)	9 (18.4)	8 (28.6)	0.299
*EDIN*	0	0	0 (0)	
*eta*	0	0	0 (0)	
*etb*	0	0	0 (0)	
*sea*	38 (49.4)	24 (49)	14 (50)	0.931
*seb*	65 (84.4)	37 (75.5)	28 (100)	0.012
*sec*	22 (28.6)	16 (32.7)	6 (21.4)	0.294
*sed*	13 (16.9)	12 (24.5)	1 (3.6)	0.041
*see*	4 (5.2)	3 (6.1)	1 (3.6)	1
*seg*	53 (68.8)	25 (51)	28 (100)	*p* < 0.001
*seh*	33 (2.9)	12 (24.5)	21 (75)	*p* < 0.001
*sei*	53 (68.8)	25 (51)	28 (100)	*p* < 0.001
*sej*	37 (48.1)	14 (28.6)	23 (82.1)	*p* < 0.001
*sek*	58 (75.3)	32 (65.3)	26 (92.9)	0.007
*sel*	50 (64.9)	24 (49)	26 (92.9)	*p* < 0.001
*sem*	45 (58.4)	23 (46.9)	22 (78.6)	0.007
*sen*	47 (61)	23 (46.9)	24 (85.7)	0.001
*seo*	50 (64.9)	24 (49)	26 (92.9)	*p* < 0.001
*sep*	10 (13)	6 (12.2)	4 (14.3)	1
*seq*	50 (64.9)	29 (59.2)	21 (75)	0.162

a The positive rates of virulence genes among CA-MRSA strains were compared to those among HA-MRSA isolates.

### Antimicrobial susceptibility

**Figure 1:**
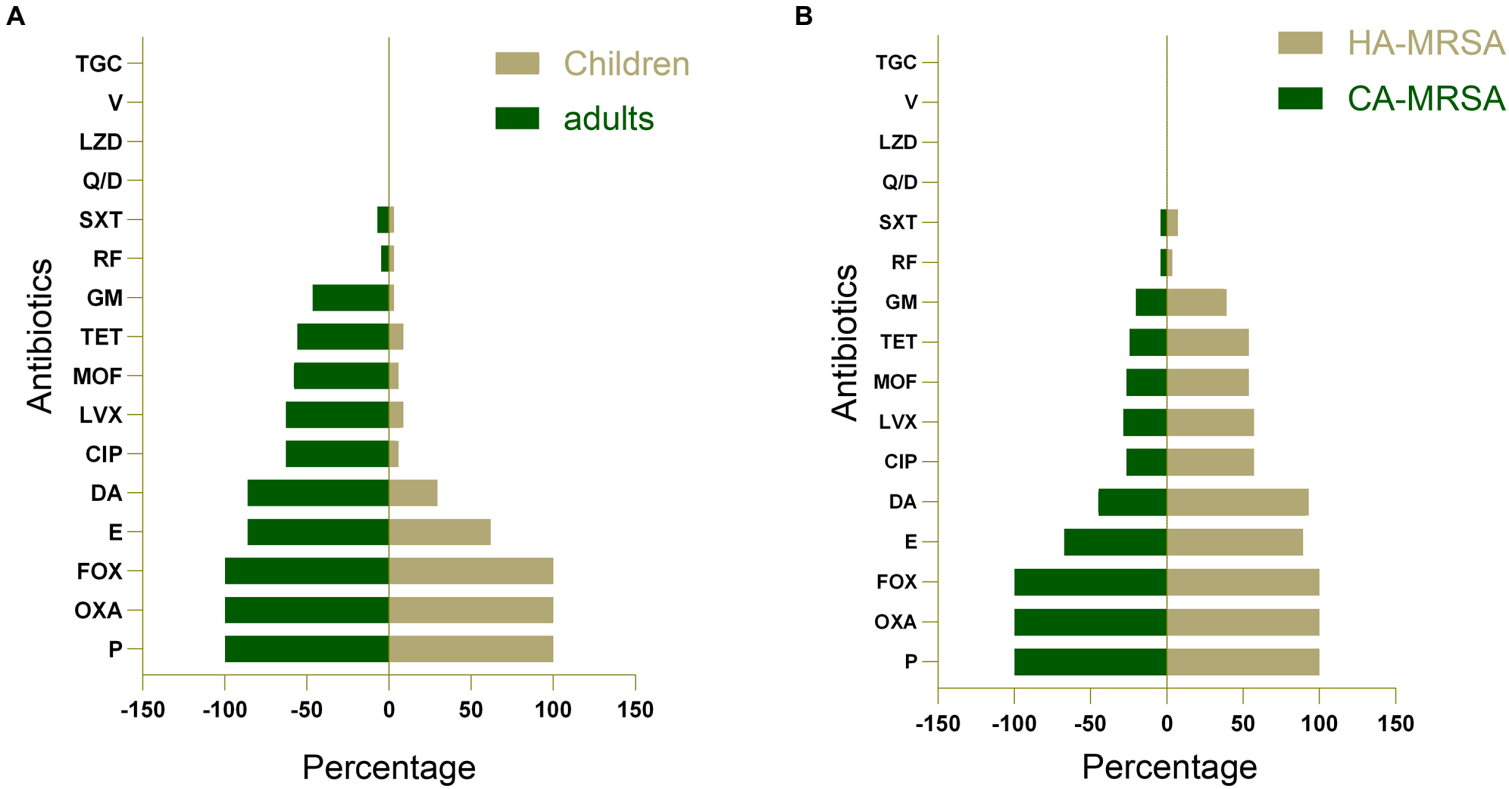
**(A)** A comparison of antimicrobial resistance profiles of methicillin-resistant *S. aureus* (MRSA) isolates obtained from adults and children. **(B)** A comparison of antimicrobial resistance profiles of community-acquired MRSA (CA-MRSA) and hospital-associated MRSA (HA-MRSA) isolates. P, penicillin; OXA, oxacillin; E, erythromycin; DA, clindamycin; CIP, ciprofloxacin; LVX, levofloxacin; MOF, moxifloxacin; TET, tetracycline; GM, gentamicin; RF, rifampicin; SXT, trimethoprim-sulfamethoxazole; Q/D, quinupristin/dalfopristin; LZD, linezolid; V, vancomycin; TGC, tigecycline; FOX, cefoxitin.

All 77 MRSA isolates were examined for antimicrobial susceptibility; their antimicrobial resistance profiles based on MLST are presented in Table 1. All strains were resistant to penicillin, oxacillin, and cefoxitin; however, they were completely susceptible to several antibiotics tested: quinupristin/dalfopristin, linezolid, vancomycin, and tigecycline. Antimicrobial resistance profiles of the adult MRSA isolates revealed that most isolates were resistant to erythromycin (86%), clindamycin (86%), ciprofloxacin (62.8%), levofloxacin (62.8%), moxifloxacin (58.1%), tetracycline (55.8%), gentamicin (46.5%), rifampicin (4.7%), and trimethoprim–sulfamethoxazole (7.0%). However, the resistance rates of pediatric MRSA isolates were 61.8% for erythromycin, 29.4% for clindamycin, 5.9% for ciprofloxacin and moxifloxacin, 8.8% for levofloxacin and tetracycline, and 2.9% for gentamicin, rifampicin, and trimethoprim–sulfamethoxazole (Supplementary Table S2). Generally, adult MRSA strains exhibited much higher resistance rates than pediatric strains (*p* < 0.05; Figure 1A). Among the children’s MRSA strains, only two (2.6%) were resistant to more than nine antibiotics, whereas almost all (50.6%) adult MRSA strains were found to be resistant to at least five antibiotics. Furthermore, 2 (2.6%) of the adult MRSA isolates were resistant to 11 antibiotics, 18 (23.4%) strains were resistant to 10 antibiotics, and 6 (7.8%) were resistant to more than 8 antibiotics. HA-MRSA strains had much higher resistance rates than CA-MRSA strains (*p* < 0.05; Figure 1B).

## Discussion

Methicillin-resistant *Staphylococcus aureus*, a virulent and difficult-to-treat “superbug,” has the potential to optimize its gene content and expression to create new strains with augmented virulence and colonization capabilities (Lakhundi and Zhang, 2018). MRSA infection is frequently associated with significant morbidity, length of hospital stay, mortality, and financial burden. MRSA BSI are a critical challenge in healthcare, accounting for a considerable percentage of nosocomial infections (Ayau et al., 2017). The potentially dangerous consequences of MRSA BSI in adult and pediatric patients make it necessary to investigate the molecular characteristics and virulence gene profiles of MRSA strains isolated from BSI to develop preventive methods against contracting and spreading MRSA in hospitals and local communities.

A recent review reported the global prevalence of CCs among clinical MRSA isolates, both as carriage and infectious isolates; the most prevalent clones worldwide were CC5 and CC8 (Lakhundi and Zhang, 2018). We obtained 43 adult and 34 pediatric MRSA strains from three hospitals to investigate their molecular characteristics. The results revealed that the largest clusters were CC59 and CC5. The most prevalent *spa* types were t032 (Ludden et al., 2015), t008, and t002 in Europe; t037 and t002 in Asia; t030 was the predominant *spa* type mainly located in China (Liu et al., 2009; Chen et al., 2010); t008, t002, and t242 in America; t020 in Australia; and t037, t084, and t064 in Africa (Asadollahi et al., 2018). Further, SCC*mec* typing was performed on 4,179 *spa* types by 41 studies, and the most prevalent SCC*mec* types were types III and II in Asia (Asadollahi et al., 2018). In China, ST239-MRSA-SCC*mec* III and ST5-MRSA-SCC*mec* II have been identified as major epidemic MRSA clones with unique geographic distributions throughout the country (Liu et al., 2009; Zhang et al., 2009). In our study, ST5/ST764-SCC*mec* II and ST59-SCC*mec* IV/V were the most prevalent clones, with the presence of minor similarities and differences. The molecular characteristics of *S. aureus* isolates differ among cities, even in China. In a study of 18 teaching hospitals in 14 Chinese cities, *spa* type t002 was the most common in Dalian (53.4%) and Shenyang (44.4%); *spa* type t037 was predominant in Shanghai (74.8%), whereas *spa* type t030 was the most common in other cities (Liu et al., 2009). The dominant types in Wenzhou were ST239 and ST188 (Yu et al., 2012), the predominant type in Shenyang and Dalian was ST5 (Liu et al., 2009), and in Chengdu, ST59 was prevalent (Tan et al., 2019). SCC*mec*-MRSA-IVa was the predominant SCC*mec* type, and specifically, ST45-MRSA-SCC*mec* IVa, an infrequent type in mainland China, was dominant in Hainan (Li et al., 2019). Among previously reported Chinese MRSA isolates, the most prevalent *spa* types were *spa* t030 and t037 (Liu et al., 2009; Chen et al., 2010). In our study, *spa* t437 was the most common type, followed by t1084 and t034. The major SCC*mec* was type IV and V. As displayed in Table 1, ST59 was the most common type, followed by ST5, ST764, and ST398. This comparison indicated that various types were prevalent and circulated in different countries or regions.

The elucidation of the difference between adult and children’s MRSA strains isolated from BSI samples is crucial; however, there have been limited studies on this comparison. ST59-MRSA-t437-IV was prevalent among children in China (Wang et al., 2016; Song et al., 2017; Yang et al., 2017). CA-MRSA pneumonia in children was largely associated with the spread of the ST59-MRSA-IV clone confined in eight Chinese hospitals countrywide (Geng et al., 2010b). In the present study, the most prevalent types of children’s MRSA isolates were ST59 and ST398 isolates, whereas ST5 and ST764 isolates were mainly derived from adult MRSA specimens. *Spa* t437 was the most predominant type, followed by t1084. The combinations of ST5-t2460, ST764-t1084, and ST59-t437 were predominant in adults, whereas ST59-t437 and ST59-t172 were predominant in children. The most common type among pediatric MRSA isolates was ST59-t437/t172-SCC*mec* IV, whereas ST5-t2460-SCC*mec* II and ST764-t1084-SCC*mec* II were the most common types among adult MRSA isolates. In a similar study conducted in South Korea, the most common *spa* type among ST5-MRSA-II isolates obtained from adult patients was t2460 (59.4%), followed by t9353 (14.4%) and t002 (8.1%) (Kim et al., 2022). Further, a study based on two children’s hospitals in Shanghai also indicated that ST59-SCC*mec* IV-t172 (34.7%) was the dominant clone in MRSA, followed by ST59-SCC*mec* IV-t437 (18.4%) (Song et al., 2017). The most prevalent SCC*mec* type of adult MRSA isolates was type II, accounting for approximately 50% of all adult MRSA isolates. Among the pediatric MRSA isolates, the most common were type IV, which was in agreement with the results reported by previous studies (Song et al., 2017; Yang et al., 2017; Table 1).

MRSA infections can be further classified into HA-MRSA and CA-MRSA infections, which differ in terms of their clinical features and molecular biology as well as their antibiotic susceptibility and treatment (Elward et al., 2009; Shahkarami et al., 2014; Lakhundi and Zhang, 2018). Some researchers have found an increase in the rate of CA-MRSA infections (3.2% in 2008 vs. 20.2% in 2019) out of all staphylococcal infections (Galper et al., 2021). Furthermore, SCC*mec* types have been reported to differ between CA-MRSA and HA-MRSA. HA-MRSA infections were usually associated with SCC*mec* types I, II, and III, whereas CA-MRSA infections were associated with SCC*mec* types IV, V, VI, VII, and VIII (International Working Group on the Classification of Staphylococcal Cassette Chromosome Elements (IWG-SCC), 2009; David and Daum, 2010). We found that the predominant types of HA-MRSA were IV, II, and III, whereas IV and V were the top two dominant types of CA-MRSA, confirming the preceding conclusions. In addition, CC59 was the more common type of CA-MRSA prevailing in Taiwan (Chen and Huang, 2014) and was the predominant type in our study. In China, the dominant epidemic CA-MRSA clone was ST59-SCC*mec* IV/V-t437 (Geng et al., 2010a); however, minor differences were noted. In our study, ST398-SCC*mec*V-t034, ST59-SCC*mec* IV-t172 and ST59-SCC*mec* IV/V-t437 were identified as the major epidemic CA-MRSA clones. In 2017, a study showed that ST59-SCC*mec* IV-t172 had replaced ST59-SCC*mec* IV-t437 as the most common clone of CA-MRSA in Shanghai (Song et al., 2017). Moreover, during 2008–2017, the prevalence of epidemic CA-MRSA ST59 and ST398 clones also increased from 1.0 to 5.8% and from 1.8 to 10.5%, respectively (Dai et al., 2019). Previous findings regarding the prevalence of ST59 were confirmed by our present data. In this study, ST59-SCC*mec* IV/V represented the most predominant clones among CA-MRSA isolates, which validates our conclusions. Other common clones were ST764/ST5-MRSA-SCC*mec* II and ST398-MRSA-SCC*mec* V.

Hospital-associated MRSA remains the most common cause with respect to multidrug resistance of hospital-associated nosocomial infection (Köck et al., 2010). A 2014 study conducted in six major Taiwanese hospitals confirmed the decreasing prevalence of ST239 and increasing prevalence of ST5 in the region (Chen et al., 2014). Presently, ST5 and ST239 are the most frequently found clones of HA-MRSA (Lakhundi and Zhang, 2018). Hospital-associated ST5-II (CC5) mainly replaced ST239-III (CC8) as the major pandemic HA-MRSA clone (Nikolaras et al., 2019). The prevalence of the predominant HA-MRSA clones ST239-t030 and ST239-t037 significantly decreased (from 20.3 to 1% and from 18.4 to 0.5% during 2008–2017, respectively); both of these clones have been replaced by the continually spreading ST5-t2460 clone, with an increase in its prevalence from 0 to 17.3% during 2008–2017(Dai et al., 2019). Consistent with these findings, we found that ST5-SCC*mec* II and CC5-SCC*mec* II-t1084 were the most predominant types among HA-MRSA clones. Furthermore, previous studies implied that among the species, some prevailing clones arose and spread throughout China (Chen et al., 2014; Dai et al., 2019).

The ability of *S. aureus* to efficaciously infect humans is primarily attributed to the expression of virulence factors, such as SE genes, PVL, and *tst*, which promote adhesion, acquisition of nutrients, and evasion of the immunologic responses of the host (Monday and Bohach, 1999). However, different regions have reported variations in the percentages of PVL-positive isolates. The detection rates of PVL were 11.1% in Taiwan, 45.2% in Guangzhou, 47.6% in Hainan, and 47.7% in Quanzhou (Wang et al., 2010; Xie et al., 2016; Li et al., 2019; Bai et al., 2021). Furthermore, a nationwide survey of MRSA isolates obtained from Chinese children at eight hospitals revealed that 40% of the isolates were PVL-positive (Geng et al., 2010b). Similarly, in our study, the presence of PVL-positive toxin was 46.8%, and the detection rate of PVL virulence factor was high, suggesting the strong toxicity of MRSA and a high pathogenic risk of MRSA invasive severe infection. Tsouklidis et al. (2020)) reported that a PVL-positive toxin was detected in more than 97% of CA-MRSA strains, but this toxin was not detected in HA-MRSA strains. In contrast, Bai et al. (2021)) found no significant difference between CA-MRSA (51.2%) and HA-MRSA (40.9%) strains in terms of the presence of PVL-positive toxin. In the present study, the positivity rates for the gene encoding PVL among the CA- and HA-MRSA isolates were 53.1 and 35.7%, respectively; therefore, the abovementioned results showing no significant difference were verified by performing further experiments (*p* = 0.142; Table 5). Consequently, PVL may no longer be recognized as a reliable marker of CA-MRSA isolates. The difference in positive rates between CA-MRSA and HA-MRSA has become indistinguishable. Increased clonal spread of PVL-positive strains has resulted in serious public health concerns that have endangered people’s health and safety over the last two decades. In addition, studies have demonstrated that PVL-positive strains resulted in high morbidity and mortality rates, spread in the hospital environment, and led to outbreaks (Zetola et al., 2005; Duman et al., 2013; Bai et al., 2021). Clinicians should pay more attention to PVL-positive strains, and continuous monitoring is necessary to prevent epidemiologic spread in the hospital environment.

Virulence gene analysis demonstrated diversity among adults and children: *see–sep* were present in all CC88 pediatric strains only; *seb–sei* were present in all adult strains; *seb–seg–sei–seo* were present in all ST5, ST59, ST15, ST45, and ST22 adult strains; and *seg–sei–sem–sen–seo* were present in different clones, including ST15, ST45, and ST22 adult MRSA isolates and ST25, ST30, ST546, and ST72 children’s MRSA strains. The frequencies of *seb*, *she*, *seg*, *sei*, *sej*, *sel*, *sem*, *sen*, and *seo* were significantly higher among the adult MRSA isolates than among the pediatric isolates (*p* < 0.05). These important genetic elements, in varying proportions, may contribute to MRSA resistance and be associated with the severity and prognosis of MRSA infection. The most prevalent SE genes were *sea* and *seb* (Ler et al., 2006). In our study, *seb* (84.4%) was the most common gene among these isolates, followed by *sea*, which accounted for 49.4% of the isolates. Song et al. (2017)) also confirmed that *seb* (39.3%) was the most frequent toxin gene, followed by *sea* (26.2%) (Song et al., 2017). Moreover, *sed* is suggested to be the second most common staphylococcal serotype associated with food poisoning worldwide (Pinchuk et al., 2010). In the present study, *sed* was significantly higher among the CA-MRSA isolates than among the HA-MRSA isolates (24.5% vs. 3.6%; *p* = 0.041; Table 5) and was only present in pediatric MRSA isolates. There was the same proportion between *sei* and *seg* in our study. Previous studies have indicated associations between *seg* and *sei*, in which the most frequent *SE* genes detected in different locations were *seg/sei*, as reported in other countries (Loncarevic et al., 2005; Kolawole et al., 2013; Ayeni et al., 2018). *seg* and *sei* were frequently encountered together because they were within the same cluster in a 3.2-kb DNA fragment (Asiimwe et al., 2017). The high co-occurrence of *seg* and *sei* found in this study was worrisome because it has been reported that *seg*, *sei*, and *ser* exhibit emetic activities (Denayer et al., 2017).

*Staphylococcus aureus* has a remarkable ability to form an adherent multilayered biofilm, which becomes resistant to antimicrobial therapy and host defenses. The development of a staphylococcal biofilm is a multifactorial process consisting of primary attachment and subsequent intercellular aggregation (Schilcher and Horswill, 2020). Primary attachment can be mediated by various adhesion genes, including *sdrC*, *sdrD*, and *sdrE*; the next accumulation into a multilayer community requires the synthesis of an exopolysaccharide called polysaccharide intercellular adhesin (PIA). PIA (Gholami et al., 2019; Mirzaei et al., 2021) is the product of the intercellular adhesion operon (icaADBC).In this study, adhesion genes were present in 83.1–100% *S. aureus* isolates. The percentages were also similar to those reported by Chen et al. (2020)) in terms of the presence of *clfA*, *icaA*, *sdrC*, *sdrD*, and *sdrE* in most isolates (Chen et al., 2020; Uribe-García et al., 2021; Zamani et al., 2022), confirming that these were the most common virulence factors in *S. aureus*, and no regional difference was noted in their distribution. This might indicate that adhesion genes were highly correlated with BSI caused by the invasion of MRSA strains. Although almost all isolates were *lukE*-positive, in which the frequencies of adult and pediatric MRSA isolates were 100 and 82.4%, respectively, none were detected as *lukM*-positive. This suggests that *lukE* is more associated with BSI than *lukM*. This phenomenon deserves further exploration. The virulence factors of *S. aureus* play an important role during pathogenesis. Although there has been a lot of research, our understanding of the specific and physiological roles of the various toxins remains far from complete. All of these factors may increase the risk of the development of more resistant and pathogenic strains with the acquisition of virulence genes, which is worthy of further exploration and research. Widespread and frequent antibiotic use amplifies environmental pools of antibiotic resistance genes and increases the likelihood of selecting a resistance event in human pathogens (Wencewicz, 2019). As for antibiotic susceptibility profiles, in general, adult MRSA strains showed much higher resistance rates to the tested antibiotics than pediatric strains in our study (*p* < 0.05), suggesting that using antibiotics frequently may induce resistance easily in adults.

This study has several limitations; for instance, the sample size is not sufficiently large, limiting the representative significance of the research. Moreover, the severity of illness at presentation and antibiotic treatment of the patients were not analyzed.

## Conclusion

In summary, *S. aureus* isolates in southern China have unique molecular characteristics and virulence gene profiles. In our study, the predominant combinations were CC5-SCC*mec* II and CC59-SCC*mec* IV, and all MRSA isolates harbored seven or more virulence genes. Those important genetic components with various subtypes and categories contribute to a better understanding of the different virulence patterns and resistance characteristics of HA-MRSA and CA-MRSA between adults and children. Our findings might provide further insight into MRSA BSI in China.

## Data availability statement

The original contributions presented in the study are included in the article/Supplementary material, further inquiries can be directed to the corresponding author.

## Author contributions

YaZ and YeZ designed the studies and contributed to manuscript revision. YaZ, YeZ, and FZ obtained the funding. RZ, XW, XW, and BD performed the experiments. RZ and KX performed the statistical analysis. RZ wrote the manuscript. XW, XW, and CJ contributed the strains. All authors have read and approved the submitted version.

## Funding

This work was supported by the National Natural Science Foundation of China (Grant Nos. 81802071 and 82170563); “123” advantageous disciplines, core technologies of the Second Affiliated Hospital of Nanjing Medical University.

## Conflict of interest

The authors declare that the research was conducted in the absence of any commercial or financial relationships that could be construed as a potential conflict of interest.

## Publisher’s note

All claims expressed in this article are solely those of the authors and do not necessarily represent those of their affiliated organizations, or those of the publisher, the editors and the reviewers. Any product that may be evaluated in this article, or claim that may be made by its manufacturer, is not guaranteed or endorsed by the publisher.

## Footnotes

^1^ https://www.spaserver.ridom.de/

^2^ https://www.ridom.de/doc/Ridom_spa_sequencing

^3^ https://pubmlst.org/

## Abbreviations

CC: clonal complex
MLST: multilocus sequence typing
MRSA: methicillin-resistant *Staphylococcus aureus*
MSSA: methicillin-susceptible *Staphylococcus aureus*
PVL: Panton-Valentine leukocidin
*S. aureus*: *Staphylococcus aureus*
SCC*mec*: staphylococcal chromosomal cassette mec
