# Supplementary material for: Molecular characterization and virulence gene profiling of methicillin-resistant Staphylococcus aureus associated with bloodstream infections in southern China
Source: Front Microbiol. 2022 Oct 17;13:1008052. doi: 10.3389/fmicb.2022.1008052 (PMC9618618; doi:10.3389/fmicb.2022.1008052)
Supplement: Supplementary file 1 [file Table_1.docx]

| **TABLE S1\|**Partial primers used in this study.. | | |
| --- | --- | --- |
| Method | Primer | Sequence |
| *mec*A | *mecAF* | 5’-GCCGTAGTTGTCGGGTTTGG-3’ |
|  | *mecAR* | 3’-GGCGGATGTGCGATTGTATTGC-5’ |
| *spa* type | *SPA113f* | 5’-TAAAGACGATCCTTCGGTGAGC-3’ |
|  | *SPA1514r* | 5’-CAGCAGTAGTGCCGTTTGCTT-3’ |
| SCC*mec* type | *β* | 5’-ATTGCCTTGATAATAGCCTTCT-3’ |
|  | *α3* | 5’-TAAAGGCATCAATGCACAAACACT-3’ |
|  | *ccrCF* | 5’-CGTCTATTACAAGATGTTAAGGATAAT-3’ |
|  | *ccrCR* | 5’-CCTTTATAGACTGGATTATTCAAAATAT-3’ |
|  | *1272F1* | 5’-GCCACTCATAACATATGGAA-3’ |
|  | *1272R1* | 5’-CATCCGAGTGAAACCCAAA-3’ |
|  | *5RmecA* | 5’-TATACCAAACCCGACAACTAC-3’ |
|  | *5R431* | 5’-CGGCTACAGTGATAACATCC-3’ |
| MLST type | *arc up* | 5’-TTGATTCACCAGCGCGTATTGTC-3’ |
|  | *arc dn* | 5’-AGGTATCTGCTTCAATCAGCG-3’ |
|  | *aro up* | 5’-ATCGGAAATCCTATTTCACATTC-3’ |
|  | *aro dn* | 5’-GGTGTTGTATTAATAACGATATC-3’ |
|  | *glp up* | 5’-CTAGGAACTGCAATCTTAATCC-3’ |
|  | *glp dn* | 5’-TGGTAAAATCGCATGTCCAATTC-3’ |
|  | *gmk up* | 5’-ATCGTTTTATCGGGACCATC-3’ |
|  | *gmk dn* | 5’-TCATTAACTACAACGTAATCGTA-3’ |
|  | *pta up* | 5’-GTTAAAATCGTATTACCTGAAGG-3’ |
|  | *pta dn* | 5’-GACCCTTTTGTTGAAAAGCTTAA-3’ |
|  | *tpi up* | 5’-TCGTTCATTCTGAACGTCGTGAA-3’ |
|  | *tpi dn* | 5’-TTTGCACCTTCTAACAATTGTAC-3’ |
|  | *yqi up* | 5’-CAGCATACAGGACACCTATTGGC-3’ |
|  | *yqi dn* | 5’-CGTTGAGGAATCGATACTGGAAC-3’ |
