# Supplementary material for: Molecular characterization and virulence gene profiling of methicillin-resistant Staphylococcus aureus associated with bloodstream infections in southern China
Source: Front Microbiol. 2022 Oct 17;13:1008052. doi: 10.3389/fmicb.2022.1008052 (PMC9618618; doi:10.3389/fmicb.2022.1008052)
Supplement: Supplementary file 2 [file Table_2.docx]

| **TABLE S2** \| Antimicrobial susceptibilities of methicillin-resistant S. *aureus* (MRSA) isolates obtained from adults and children. | | | | |
| --- | --- | --- | --- | --- |
|  | *S. aureus*，  n=77, n(R^a^ %) | adults (n = 43),  n=43,n(R^a^ %) | children (n = 34),  n=34,n(R^a^ %) | *P*-value^b^ |
| P | 77(100) | 43(100) | 34(100) |  |
| OXA | 77100) | 43(100) | 34(100) |  |
| E | 59(76.6) | 37(86) | 21(61.8) | 0.014 |
| DA | 47(61) | 37(86) | 10(29.4) | *P*<0.001 |
| CIP | 29(37.7) | 27(62.8) | 2(5.9) | *P*<0.001 |
| LVX | 30(39) | 27(62.8) | 3(8.8) | *P*<0.001 |
| MOF | 28(36.4) | 25(58.1) | 2(5.9) | *P*<0.001 |
| TET | 27(35.1) | 24(55.8) | 3(8.8) | *P*<0.001 |
| GM | 21(27.3) | 20(46.5) | 1(2.9) | *P*<0.001 |
| RF | 3(3.9) | 2(4.7) | 1(2.9) | 1 |
| SXT | 4(5.2) | 3(7) | 1(2.9) | 0.783 |
| Q/D | 0 | 0 | 0 |  |
| LZD | 0 | 0 | 0 |  |
| V | 0 | 0 | 0 |  |
| TGC | 0 | 0 | 0 |  |
| FOX | 77(100) | 43(100) | 34(100) |  |

^a^R = resistance. ^B^ The resistance rates of antimicrobials among adult strains were compared to those among children isolates. penicillin (P), oxacillin (OXA), erythromycin (E), clindamycin (DA), ciproflfloxacin (CIP), levoflfloxacin (LVX), moxiflfloxacin (MOF), tetracycline (TET), gentamicin (GM), rifampicin (RF), trimethoprim-sulfamethoxazole (SXT), quinupristin/dalfopristin (Q/D), linezolid(LZD), vancomycin (V), tigecycline (TGC) , and Cefoxitin (FOX).
