# Supplementary material for: Molecular characterization and virulence gene profiling of methicillin-resistant Staphylococcus aureus associated with bloodstream infections in southern China
Source: Front Microbiol. 2022 Oct 17;13:1008052. doi: 10.3389/fmicb.2022.1008052 (PMC9618618; doi:10.3389/fmicb.2022.1008052)
Supplement: Supplementary file 3 [file Table_3.docx]

| **TABLES3** \| Antimicrobial susceptibilities of community-acquired methicillin-resistant S. *aureus* (CA-MRSA) and hospital-associated MRSA (HA-MRSA) isolates. | | | | |
| --- | --- | --- | --- | --- |
|  | *S.aureus，*  n=77,n(R^a^ %) | CA-MRSA  n=49,n(R^a^ %) | HA-MRSA  n=28,n(R^a^ %) | *P*-value^b^ |
| P | 77(100) | 49(100) | 28(100) |  |
| OXA | 77100) | 49(100) | 28(100) |  |
| E | 59(76.6) | 33(67.3) | 25(89.3) | 0.032 |
| DA | 47(61) | 22(44.9) | 26(92.9) | *P*<0.001 |
| CIP | 29(37.7) | 13(26.5) | 16(57.1) | 0.008 |
| LVX | 30(39) | 14(28.6) | 16(57.1) | 0.013 |
| MOF | 28(36.4) | 13(26.5) | 15(53.6) | 0.018 |
| TET | 27(35.1) | 12(24.5) | 15(53.6) | 0.01 |
| GM | 21(27.3) | 10(20.4) | 11(39.3) | 0.074 |
| RF | 3(3.9) | 2(4.1) | 1(3.6) | 1 |
| SXT | 4(5.2) | 2(4.1) | 2(7.1) | 0.961 |
| Q/D | 0 | 0 | 0 |  |
| LZD | 0 | 0 | 0 |  |
| V | 0 | 0 | 0 |  |
| TGC | 0 | 0 | 0 |  |
| FOX | 77(100) | 49(100) | 28(100) |  |

^a^R = resistance. ^B^ The resistance rates of antimicrobials among adult strains were compared to those among children isolates. penicillin (P), oxacillin (OXA), erythromycin (E), clindamycin (DA), ciproflfloxacin (CIP), levoflfloxacin (LVX), moxiflfloxacin (MOF), tetracycline (TET), gentamicin (GM), rifampicin (RF), trimethoprim-sulfamethoxazole (SXT), quinupristin/dalfopristin (Q/D), linezolid(LZD), vancomycin (V), tigecycline (TGC) , and Cefoxitin (FOX).
